# Investigating the effect of Integrated Educational Program on the Quality of Life among Cancer Patients: A Clinical Trial Study

**DOI:** 10.31557/APJCP.2019.20.11.3457

**Published:** 2019

**Authors:** Maryam Bayati, Shahram Molavynejad, Noorollah Tahery, Bahman Cheraghian

**Affiliations:** 1 *Nursing Care Research Center in Chronic Diseases, School of Nursing and Midwifery, *; 3 *Department of Epidemiology and Biostatistics, School of Health, Ahvaz Jundishapur University of Medical Sciences, Ahvaz, *; 2 *Abadan University of Medical Sciences, Abadan, Iran. *

**Keywords:** Patient education, quality-of-life, cancer, neoplasm

## Abstract

**Background:**

cancer is one of the most common causes of death around the world. The process of this disease and the resulting complications reduce the quality of life of cancer patients. Taking the necessary measures for improving the quality of life of these patients seems to be essential. This study was performed to investigate the effect of integrated educational program on the quality of life of cancer patients.

**Methods:**

in this clinical trial study, 64 patients hospitalized in the specialized cancer hospital affiliated with Ahvaz Jundishapur University of Medical Sciences, Iran, were selected according to the inclusion criteria. Then, through blocked randomization method, they were assigned into intervention and control groups. The intervention group received the necessary trainings over four 60-min sessions (one session per week). The data collection in this study included demographic questionnaire and quality-of-life questionnaire of cancer patients (QLQ-C30). The quality of life was examined before the training as well as one and two months after the training. The data were analyzed by SPSS 20. Independent t-test was used to compare the means of the life quality dimensions of the studied groups.

**Results:**

all of the functional dimensions [physical, role function, emotional, cognitive, social(P≤0.05)] and symptomatic [fatigue, nausea and vomiting, pain, dyspnea, sleep disorders, diminished appetite, constipation, and diarrhea (P≤0.05)] of the quality of life of the intervention group increased significantly one and two months after running the integrated educational program.

**Conclusion:**

integrated training causes improved symptoms and enhanced quality of life in cancer patients. Thus, it is recommended that integrated training be conducted alongside the routine care of cancer patients. This can improve the therapeutic outcomes, and also highlights the important role of nurses as well as nursing cares.

## Introduction

Cancer is one of the main health problems around the world and is the second leading cause of death in the United States (Siegel et al., 2018). Cancer is the third leading cause of death in Iran (Majidi et al., 2017). The annual incidence of cancer in Iran was reported to be 158/100,000 people (including skin cancer) and 143,000 people (regardless of skin cancer) (Ahmadi et al., 2018). Iran is one of the developing countries, where factors including industrialization, environmental changes, and lifestyle contribute to increased incidence of cancer in this country (Almasi et al., 2015; Rohani-Rasaf et al., 2013).

Throughout the different stages of cancer treatment, various modes of treatment including surgery, radiotherapy, and chemotherapy may be used. Complications including water and electrolyte disorder, anemia, vomiting, nausea, probability of bleeding, stomatitis, diminished appetite, disordered nutrition, problem in defecation, fatigue, pain, sleep disorders, worrying about the future of the family members, fear from death, depression, and disorders in the mental image can emerge following these treatments, which in turn overshadow all dimensions of quality of life and longevity of the patients (Miladinia et al., 2018; Miladinia et al., 2017; Rueda et al., 2011; Talaiezadeh et al., 2013; Vergara et al., 2013; Wintner et al., 2013; Gheyasi et al., 2019). 

One of the important issues that has been of great interest in recent years about patients with cancer is the quality of life. By measuring the quality of life, one can examine the problems of patients recovered from cancer and the quality of cares offered. It is also possible to compare the advantages and disadvantages of treatments and cares, and screen the patients at risk of mental and social problems as well as other physical complications (Doumit et al., 2010). 

Training patients and their families is one of the important nursing activities, and can determine the difference between success and failure in adaptation with the disease (Wingard, 2005). Nurses play a significant role in making the patients’ lives positive through training and sustainable changes in their lives (Syx, 2008). One of the methods widely used today is integrated education. Various studies have shown that integrated training in health professions causes increased awareness and learning as well as reinforced training and clinical skills (Liu and Peng, 2016; Rowe et al., 2012). Today, integrated education emphasizes various and extensive use of learning methods including face-to-face learning, group learning, and individual learning as a new mechanism with the aim of applying a proper and suitable combination for every learning problem (Amirmohseni et al., 2016). It is recommended that group training should be carried out when there is a high number of patients and a low number of nursing staff. According to patients, group training is helpful in guiding them to perform proper self-care behaviors. However, some researchers believe that this training method is usually brief and does not provide patients with sufficient knowledge. Therefore, it is recommended that integrated education should be used to overcome the drawbacks of group training. In integrated education, educational videos are used to overcome the drawbacks of group training. Video training has its own advantages and plays a complementary role in educational methods (Baraz et al., 2010). The advantages of video training include the ability to create abundant information storage, information continuity, no anxiety during training, and addition of new information to previous content. Moreover, patients can learn more through simultaneous use of audio and video and various scenes of educational films (Baraz et al., 2008). Therefore, training through combining video and speech can improve patients’ learning. In comparison with traditional training, combined training facilitates social interactions and communications, increases the effectiveness of knowledge, and provides lifelong learning ( Baraz et al., 2008; Rovai and Jordan, 2004). In other studies, only one type of educational method such as lecture, in person consultation (Bahrami and Farzi, 2014; Kurtz et al., 2007), telephone consultation (Sherman et al., 2012), group training (Reif et al., 2013), individual training (Sajjad et al., 2016) has been used. However, in some studies, training has been given only in special areas including healthy nutrition (Mohammadi et al., 2013), fatigue management (Reif et al., 2013; Wangnum et al., 2013), and management of pain (Lovell et al., 2014; Yildirim et al., 2009). In addition to, integrated educational program in cancer patients has rarely been studied. This study was performed to investigate the effect of integrated educational program on the quality of life of cancer patients in Oncology Ward at Ahvaz Shafa Hospital.

## Materials and Methods

This clinical trial study was carried out at specialized cancer hospital affiliated with Ahvaz Jundishapur University of medical sciences, Ahvaz, Iran. A convenience sample was recruited. The sample size estimate was based on 80% power for detecting a clinically significant difference of 3.0 points between the 2 groups (Miladinia et al., 2017) ; the SD was assumed to be 4, with a significance level of α = .05. By assuming a 10% loss to follow-up rate, we needed to randomize 32 participants to each group. They were then allocated into two groups of intervention (n=32) and control (n=32) through blocked randomization method. Thereafter, the intervention group was divided into two 11- and one 10-person groups randomly.

The inclusion criteria included having cancer (breast, prostate, and colon), age≥16, and stage≤3 (The subjects were selected among patients with stages I to III cancer to be able to better cooperate with the researcher and take part in the training classes, while patients at stage IV are usually ill and cannot take part in the classes), background of radiotherapy and chemotherapy courses (at least twice), literacy, and ability to work with computer. On the other hand, the exclusion criteria included altered disease stage in the course of the treatment, entering the end stage phase, having a diagnosed cognitive or mental disorder including depression, schizophrenia, dementia, psychosis, bipolar disorder, as well as unwillingness to continue the cooperation, and finally death of the patient.

*Collection instrument*

The data collection instrument in this study included demographic questionnaire (age, gender, and marital status, level of education, occupation, supportive resources and number of children). Another instrument was quality of life questionnaire of cancer patients (QLQ-C30). This questionnaire includes 30 items in five functional scales including: physical (5 items), role function (2 items), emotional (2 items), and cognitive (4 items), and social (2 items), and 8 areas of symptoms (fatigue, nausea and vomiting, pain, dyspnea, sleep disorder, diminished appetite, constipation, diarrhea). In this questionnaire, four-degree scale (never, little, much, and very much) was used to measure the responses. Except for the items related to the general health status and quality of life which had a seven-degree scale ranging from 1 (very little) to 7 (excellent). A higher score in the functional areas suggested better status of the person in that area (a score of 100 represented the best health status, while zero score showed the worst health conditions). However, regarding the area of symptoms, a higher score represented more symptoms and problems of the disease in the person (100 suggested undesirable status, while zero represented desired conditions). The Persian version of this questionnaire has been confirmed as reliable and valid by European organization for research and treatment of cancer (Safaee at al., 2008). The reliability and validity of QLQ-C30 questionnaire have been evaluated by Iranian researchers, showing a suitable validity and reliability (73-96%) (Montazeri et al., 1999; Safaee and Moghim Dehkordi, 2007). In the present study, the reliability of the tool was measured as .87 (Cronbach’s α coefficient).

*Intervention*

Before the intervention, demographic information questionnaire and quality of life questionnaire C-30 were provided to both groups, and their information was registered (pretest). In order to assess patient needs, a 20-item questionnaire was developed and provided for the patients and their knowledge about important aspects of care such as the adverse effects of chemotherapy on various body organs, diet, and medicines was evaluated. After analyzing the questionnaire, the care needs of the patients were identified and the training program was designed based on the specified needs. 

Educational content was approved by a dietitian specialist, cancer specialist, cancer nurse and psychologist. Thereafter, based on the needs analysis, the necessary trainings were given to the patients in the treatment group, and an educational film was provided to them between the sessions. After one month, the quality of life of these patients (as in person follow-up and using C-30 questionnaire) was measured again (posttest one month after training). Further, one month later (posttest two months after training) the patients’ quality of life was measured again with using the mentioned questionnaire. The trainings given based on needs analysis were presented in group during four 60-min sessions with a one-week interval. Each session began with a brief reception and talk about the objective of that session, and 20% of the time of each session was dedicated to free discussion and emotional support from each other, and experiencing the personal experiences. In the first session, after explaining the research objectives, about 30 minutes was dedicated to spiritual cares to attract cooperation of all patients in implementing the effective treatments on quality of life. Another 15 minutes was allocated to cares of skin and mucous complications, and 30 min was dedicated to training the suitable diet for cancer patients. Finally, 15 min was left for question and answer session. In the second session, about 20, 20, 30, and finally 20 min were dedicated to the cares about dispelling fatigue and sleep pattern; cares for loss of appetite; diet; and question and answer, respectively. In this research, group training method was used in the presence of one active family member of the patient through a projector and audiovisual equipment. Also, an educational film was provided to the patients between the educational sessions. The necessary trainings were organized by the researcher in 10-12 person classes. The fact that the patients were present among other people who had also cancer like them, caused psychological alleviation of patients and their improved cooperation in care and treatment. Further, presence of one active family member led to improved cares. It also resulted in limiting the number of sessions due to reduced unwillingness and fatigue among the patients, where their limited number of sessions was compensated for to some extent by presenting the educational film. In other words, integrated education using a combination of several methods can cause increased level of the effect of education on the quality of life of patients. The training program was conducted for the intervention group in a classroom out of the ward and attempts were made to prevent any contact between the intervention and the control groups. Moreover, during the intervention period, a nurse stayed with the patients in the control group to maintain their conditions and to take sufficient care of them (social attention) and encourage them to express their feelings and worries concerning hospitalization. The patients in the control group were assured that the care program would be performed for them in a session and the contents of the program would be provided in the form of a booklet after conducting the training program for the intervention group

*Statistical analysis*

The data were analyzed by SPSS 20. Data normality was examined by Kolmogrov-Smirnov test. Independent t-test was used to compare the mean of quality of life dimensions of the studied groups. Further, chi-square test was employed to investigate homogeneity of the two groups in terms of demographic variables. The significance level of the tests was considered 5%.

*Ethical considerations*

This research has been registered with the ethics code of ajums.REC.1393.327 in ethics committee of Ahvaz Jundishapur University of Medical Sciences, Ahvaz, Iran. First, the objectives of this research were explained to the participants completely, and if they consented, written consent form was taken from them. After the last stage of data collection, to follow ethical principles, the educational pamphlet was also provided to the control group, and their questions were also answered. This study was registered in the Iranian website of Registry of Clinical Trial under the code of IRCT2015012720834N1. 

## Results

There was no statistically significant difference between the groups in terms of age, gender, marital status, occupation, number of children, and individual support (p>0.05). According to the results, 75% and 53% of the patients were male in the intervention and control groups, respectively. A total of 53.1% of the patients in both groups were in the age range of 25-50 years; 96.6% and 93.8% of the patients were married in the intervention and control groups, respectively; 62.5% and 56.3% of the patients were housewives in the intervention and control groups, respectively; and 96.9% of the patients in both groups lived with their family (Table 1).

Table 2 indicates the mean scores of dimensions of functional indicators of quality of life (physical, role, emotional, mental, and social) as well as the general health status and quality of life in the intervention group (before training, one month after training, two months of the training). The mean scores of all dimensions of the functional indicators of quality of life (physical, role, emotional, mental, and social) increased significantly in the treatment group one and two months after the training (p<0.05).

Table 3 indicates the mean scores of the dimensions of functional indicators of quality of life (physical, role, emotional, mental, and social) as well as a general health status and quality of life in the control group (before the study, one and two months after). The mean scores of all of these dimensions did not change significantly and even increased in the control group one and two months after. 

Table 4 provides the mean scores of symptomatic indicator of quality of life (fatigue, nausea, pain, dyspnea, insomnia, anorexia, constipation, and diarrhea) in the intervention group (before the training, one month after training, two months after training). The mean scores of all of these indicators increased significantly in the treatment group one and two months after the training (p<0.05).

Table 5 shows the mean scores of symptomatic indicator of quality of life (fatigue, nausea, pain, dyspnea, insomnia, anorexia, constipation, and diarrhea) in the control group (before the training, one month after training, two months after training). The mean scores of all of these indicators changed slightly and even decreased after two months in the control group.

**Table 1:** Demographic Characteristics of the Study Subjects

Variables	Group		P value
	Intervention	Control	
Sex			
Male	75%	53%	p=0.11
Female	25%	49%	
Age			
25-50	53.10%	53.10%	p=0.61
50-75	46.90%	43.80%	
> 75	0%	3.10%	
Marital status			
Married	96.60%	93.80%	p=0.67
Single	9.40%	6.30%	
Job			
Housekeeper	62.50%	56.30%	
Employee	12.50%	9.40%	p=0.74
Laborer	6.30%	28.10%	
Retired	18.80%	6.30%	
Number of children			
0	12.50%	9.40%	
1-2	25%	34.40%	p=0.82
3-5	46.90%	37.50%	
5<	15.60%	18.80%	
Individual supporter			
Wife, family, children	96.90%	96.90%	
Friends and relatives	3.10%	3.10%	p=0.74
Education			
Under Diploma	46.90%	50%	
Diploma	46.90%	40%	p=0.86
University education	6.20%	10%	

Chi-square test showed that no statistically significant difference between the groups in terms of age, gender, marital status, occupation, number of children, and individual support (p>0.05).

**Table 2:** The Mean Score Functional Scales and Global QoL in Intervention Group

	Baseline	after 1 month	after 2 month		P
Dimension	Mean (SD)	Mean (SD)	Mean (SD)	t statistic (df)	
physical	64.80 (17.08)	70.25 (19.51)	75.22 (18.94)	3.28 (62)	0.02٭
role	72.90 (19.78)	81.07 (22.11)	86.79 (20.73)	2.74 (62)	0.008٭
emotional	51.12 (20.94)	61.99 (22.97)	64.84 (22.79)	2.51 (62)	0.015٭
Cognitive	53.08 (19.13)	59.35 (20.13)	65.43 (21.90)	2.4 (62)	0.019٭
social	67.38 (18.90)	73.73 (21.85)	78.64 (20.66)	2.27 (62)	0.026٭
global QoL	51.42 (17.65)	60.35 (19.11)	64.38 (19.32)	2.8 (62)	0.007٭

٭, statistically significant

**Table 3:** The Mean Score Functional Scales and Global QoL in Control Group

	Baseline	after 1 month	after 2 month		P
Dimension	Mean (SD)	Mean (SD)	Mean (SD)	t statistic (df)	
Physical	62.81 (18.27)	61.78 (19.88)	60.76 (17.07)	0.46 (62)	0.64
Role	69.88 (21.77)	70.01 (20.76)	67.99 (21.76)	0.35 (62)	0.73
Emotional	50.13 (19.93)	50.20 (18.92)	49.11 (17.91)	0.22 (62)	0.83
Cognitive	51.09 (16.12)	51.05 (17.12)	48.88 (16.08)	0.55 (62)	0.59
Social	65.90 (17.89)	65.99 (18.88)	63.80 (17.87)	0.47 (62)	0.64
Global QoL	49.51 (17.65)	49.61 (17.65)	47.01 (17.65)	0.57 (62)	0.57

**Table 4:** The Mean Score Symptoms Scale of QoL in Intervention Group

Symptom	Baseline	after 1 month	after 2 month	P
	Mean (SD)	Mean (SD)	Mean (SD)	
Fatigue	58.43 (17.91)	48.91 (19.12)	46.21 (18.15) 2.71	0.009٭
Nausea	33.65 (20.86)	28.38 (21.61)	25.58 (19.62) 1.59	0.12
pain	37.28 (21.71)	31.70 (19.80)	29.01 (20.81) 1.56	0.13
Dyspnea	18.42 (10.81)	10.29 (9.92)	8.30 (5.57) 4.71	>0.001٭
insomnia	45.67 (21.06)	34.87 (20.99)	30.01 (19.05) 3.12	0.003٭
Appetite loss	61.56 (21.81)	51.85 (20.74)	46.93 (18.82) 2.92	0.005٭
Constipation	35.47 (22.11)	29.19 (19.87)	25.22 (17.83) 2.04	0.045٭
Diarrhea	19.57 (11.76)	14.51 (9.76)	11.04 (3.93) 3.89	>0.001٭

٭, statistically significant

**Table 5:** The Mean Score Symptoms Scale of QoL in Control Group

Symptom	Baseline	after 1 month	after 2 month	P
	Mean (SD)	Mean (SD)	Mean (SD)	
Fatigue	57.12 (19.90)	57.55 (19.89)	57.01 (18.78)	0.56
Nausea	31.89 (18.89)	30.87 (17.32)	30.65 (18.60)	0.67
pain	36.25 (20.54)	35.98 (19.68)	36.31 (19.78)	0.65
Dyspnea	16.55 (9.78)	15.05 (8.78)	14.21 (8.54)	0.81
insomnia	46.38 (21.05)	46.65 (19.04)	45.76 (19.06)	0.77
Appetite loss	62.63 (22.03)	61.99 (21.78)	62.01 (20.79)	0.58
Constipation	34.05 (19.23)	34.43 (18.20)	33.95 (18.21)	0.66
Diarrhea	20.37 (11.75)	20.21 (10.75)	19.89 (10.74)	0.64

## Discussion

The present study aimed to investigate the effect of combined training program on the quality of life of cancer patients. The results of this study indicated that all functional (physical, role, emotional, social, and mental) dimensions of quality of life of patients in the treatment group one and two months after the integrated educational program improved significantly. Because few studies have been conducted on the effectiveness of integrated education in patients with cancer, the results in hand are compared with other studies. Shahsavari et al., (2015) stated that training self-care enhances the total quality of life as well as physical, social, mental, and emotional dimensions in cancer patients. In the study by Mohammadi et al., (2013), it was found that training healthy diet to patients with breast cancer causes improved total quality of life as well as social, emotional, mental and role dimensions in them. Sherman et al., (2012) stated that psychological training and telephone consultation for women with cancer enhance their social, emotional, and physical adaptation. Bahrami et al., (2014) concluded that supportive educational program improves total quality of life as well as mental and physical dimensions of cancer patients and their companions. In his study, Sajjad et al., (2016) concluded that individual training of cancer patients along with emotional support causes improved emotional and physical dimensions of quality of life in these patients. Hsu et al., (2010) concluded that emotional and information consultation causes improved mental image and diminished anxiety and emotional distress in patients with breast cancer under mastectomy.

In this study, it was also found that symptomatic scales (fatigue, nausea, pain, dyspnea, insomnia, anorexia, constipation, and diarrhea) of the patients in the intervention group 1 and two months after the integrated educational program improved significantly. On the other hand, all of the symptomatic scales of the quality of life of the patients in the control group 1 and two months after the not change significantly and even decreased in some cases. Since nurses do not have enough time to educate the patient, so when the training program was designed by the researchers and was implemented for patients, all patients were very enthusiastic about the program and this made the program effective.

Kurtz et al., (2007) concluded that training control of symptoms in cancer patients undergoing chemotherapy for the first time causes improved and mitigated symptoms including pain, fatigue, and insomnia in them. Sahin and Erguney (2016) noted that training management and control of symptoms to cancer patients undergoing chemotherapy results in a significant reduction in symptoms including nausea, vomiting, anxiety, fatigue, and sleep disorders. Mollaoglu and Erdogan (2014) concluded that implementing a well-planned educational model in patients undergoing chemotherapy significantly decreases symptoms including nausea, vomiting, constipation, pain, oral and pharyngeal problems, problems of the skin and nails, anorexia, weight changes, anxiety, fatigue, and sleep disorders. Wangnum et al., (2013) concluded that multidimensional educational program mitigates fatigue in patients with lung cancer undergoing chemotherapy. Rief et al., (2013) also stated that group training method mitigates fatigue in patients with cancer. The training duration in this study was two 90-min sessions with one-week interval. Further, presence of one active family member caused improved cares, and limiting the number of sessions caused decreased unwillingness and fatigue in patients. By presenting the educational film, the limitation of number of sessions was compensated for some extent.

In conclusion, integrated education causes improved symptoms and enhanced quality of life in patients with cancer. As the duty of constant care for cancer patients is on the shoulder of nurses and training patient is one of the important duties of nurses, it is recommended that the procedure of this educational program be provided to the nursing personnel of oncology ward of hospitals, in order to help cancer patients, live better. In other words, if integrated education (based on the patients’ needs) is given alongside the routine care to cancer patients, it can improve therapeutic outcomes and also highlight the role of nurses as well as nursing cares. This strategy is better to be enforced as a standard guideline for nursing care of patients with cancer who have experienced chemotherapy, radiotherapy, and complications of cancer. It is also suggested that the long-term effect of this educational program be investigated with a larger sample in future studies.
